# New Insights From Imputed Whole-Genome Sequence-Based Genome-Wide Association Analysis and Transcriptome Analysis: The Genetic Mechanisms Underlying Residual Feed Intake in Chickens

**DOI:** 10.3389/fgene.2020.00243

**Published:** 2020-04-03

**Authors:** Shaopan Ye, Zi-Tao Chen, Rongrong Zheng, Shuqi Diao, Jinyan Teng, Xiaolong Yuan, Hao Zhang, Zanmou Chen, Xiquan Zhang, Jiaqi Li, Zhe Zhang

**Affiliations:** Guangdong Provincial Key Lab of Agro-Animal Genomics and Molecular Breeding, College of Animal Science, South China Agricultural University, Guangzhou, China

**Keywords:** imputed WGS data, GWAS, transcriptome analysis, feed efficiency, chickens

## Abstract

Poultry feed constitutes the largest cost in poultry production, estimated to be up to 70% of the total cost. Moreover, there is pressure on the poultry industry to increase production to meet the protein demand of humans and simultaneously reduce emissions to protect the environment. Therefore, improving feed efficiency plays an important role to improve profits and the environmental footprint in broiler production. In this study, using imputed whole-genome sequencing data, genome-wide association analysis (GWAS) was performed to identify single-nucleotide polymorphisms (SNPs) and genes associated with residual feed intake (RFI) and its component traits. Furthermore, a transcriptomic analysis between the high-RFI and the low-RFI groups was performed to validate the candidate genes from GWAS. The results showed that the heritability estimates of average daily gain (ADG), average daily feed intake (ADFI), and RFI were 0.29 (0.004), 0.37 (0.005), and 0.38 (0.004), respectively. Using imputed sequence-based GWAS, we identified seven significant SNPs and five candidate genes [MTSS I-BAR domain containing 1, folliculin, COP9 signalosome subunit 3, 5′,3′-nucleotidase (mitochondrial), and gametocyte-specific factor 1] associated with RFI, 20 significant SNPs and one candidate gene (inositol polyphosphate multikinase) associated with ADG, and one significant SNP and one candidate gene (coatomer protein complex subunit alpha) associated with ADFI. After performing a transcriptomic analysis between the high-RFI and the low-RFI groups, both 38 up-regulated and 26 down-regulated genes were identified in the high-RFI group. Furthermore, integrating regional conditional GWAS and transcriptome analysis, ras-related dexamethasone induced 1 was the only overlapped gene associated with RFI, which also suggested that the region (GGA14: 4767015–4882318) is a new quantitative trait locus associated with RFI. In conclusion, using imputed sequence-based GWAS is an efficient method to identify significant SNPs and candidate genes in chicken. Our results provide valuable insights into the genetic mechanisms of RFI and its component traits, which would further improve the genetic gain of feed efficiency rapidly and cost-effectively in the context of marker-assisted breeding selection.

## Introduction

Poultry feed consistutes the largest cost of poultry production, estimated to be up to 70% of the total cost (Willems et al., 2013). Moreover, there is pressure on the poultry industry to increase production to meet the protein demand of humans and simultaneously reduce emissions to protect the environment. Therefore, improving feed efficiency (FE) plays an important role to improve the profits and the environmental footprint in broiler production. Residual feed intake (RFI) is defined as the difference between actual and expected feed intake required for animal maintenance and growth (Chambers et al., 1963). It has been widely used in the genetic improvement of FE in livestock since it has superior sensitivity and accurateness in measuring feed efficiency. The RFI methodology separates the broiler breeder energy efficiency into two components: systematic sources of variation related to in individual maintenance and all other sources of variation in energy efficiency (the residual) (Romero et al., 2009). Compared with the genetic improvement of feed conversion ratios, the improvement of RFI may have a simultaneous positive effect on productivity and feed efficiency (Aggrey et al., 2010). In addition, RFI has a moderate heritability in broilers and responds to selection (Pakdel et al., 2005; Aggrey et al., 2010; Zhang et al., 2017). Although the traditional selection for RFI has made substantial genetic gain, the genetic mechanisms are still unclear (Tallentire et al., 2016). Therefore, the genetic dissection of RFI and its component traits [average daily gain (ADG) and average daily feed intake (ADFI)] would further improve the genetic gain of feed efficiency rapidly and cost-effectively.

Over the past decade, the genome-wide association analysis (GWAS), based on single nucleotide polymorphism (SNP) chip data to identify the genetic mechanisms of FE, has been widely implemented in livestock, especially in cattle (Seabury et al., 2017; Higgins et al., 2018; Schweer et al., 2018) and pig (Do et al., 2014; Bai et al., 2017; Horodyska et al., 2017). These GWAS studies revealed many candidate genes and provided useful information for genomic breeding programs to select more efficient animals in livestock. However, the RFI-related GWAS studies are still scarce in chicken. Yuan et al. (2015) performed a GWAS using 600 K SNP array and identified a haplotype block on GGA27 harboring a significant SNP (rs315135692) associated with RFI. Also, using GWAS with 600 K SNP array, our previous study showed that the effective SNPs related with RFI were located in a 1-Mb region (16.3–17.3 Mb) of GGA12 but did not identify causal variant SNPs or genes associated with RFI (Xu et al., 2016). The poor power of our previous GWAS study may be due to the small population size and lower maker density. Nowadays, it is possible to perform GWAS with whole-genome sequencing (WGS) data with the rapid development of high-throughput sequencing technology. Compared with SNP chip data, WGS data would cover all SNPs including causal mutations. Thus, performing GWAS using WGS data is expected to improve the power of test efficiency and identify the causal mutations of complex traits, and this expectation has been confirmed in dairy cattle populations (Daetwyler et al., 2014). However, sequencing 1000s of individuals of interest is still too expensive. Hence, it is an attractive and less expensive approach to obtain WGS data using genotype imputation (Li et al., 2009).

In this study, WGS data were obtained by a two-step imputation approach (from 55 to 600 K and then imputed to WGS) using a combined reference panel. Using imputed WGS data, GWAS was performed to identify SNPs and genes associated with RFI and its component traits. In addition, a transcriptomic analysis was performed to identify differentially expressed genes (DEGs) between high- and low-RFI groups to validate the candidate genes of RFI from the GWAS results and give biological evidence to candidate genes. The aims of this study were to pinpoint the associated loci and genes that contribute to the phenotypic variability in feed efficiency and provide valuable insights into the genetic mechanisms of RFI.

## Materials and Methods

### Population and Phenotyping

A chicken population derived from a yellow-feather dwarf broiler breed that was maintained for 25 generations by Wens Nanfang Poultry Breeding, Co., Ltd. (Xinxing, China) was used in this study. This population includes 1,600 birds (800 males and 800 females) and was the third batch of the 25th generation of this chicken population. These birds came from a mixture of full-sib and half-sib families from mating 30 males and 360 females of the 24th generation. After hatching, all birds were maintained in a closed building under controlled environmental conditions and provided with a standard diet until they were 4 weeks of age. The chickens were then randomly assigned to six pens by gender (three pens for males and three pens for females) for growth performance testing from 5 to 13 weeks of age. They received food and water *ad libitum* during all stages. Finally, the remaining 1,338 birds were slaughtered at 91 days of age for carcass trait recording. ADG and ADFI per individual were calculated for the period from 45 to 84 days. The RFI was calculated as follows:

RFI=ADFI-(b0+b1×MMBW+b2×ADG)

where b0 was the intercept, MMBW is mid-test body weight (MBW raised to the power of 0.75), and the MBW was the predicted body weight on day 21 of the test. b1 and b2 are the partial regression coefficients for MMBW and ADG, respectively. The descriptive statistics of the analyzed traits could be found in Table 1. For more details about this population, please refer to Xu et al. (2016) and Zhang et al. (2017).

**TABLE 1:** Basic descriptive statistics of the analyzed traits.

Trait^1^	*N*	Mean	*SD*	C.V.	Minimum	Maximum	*W*^2^	*P*-value
ADG, g/d	626	28.95	4.87	16.82%	4.66	44.65	0.99	0.003
ADFI, g/d	626	108.59	14.36	13.22%	69.79	155.92	1.00	0.054
RFI, g	626	0	8.83	−	−32.05	26.00	0.99	0.003

*^1^These traits were average daily gain (ADG), average daily feed intake (ADFI), and residual feed intake (RFI). ^2^*W* means Shapiro–Wilk statistic.*

### Genotyping, Genotype Imputation, and Quality Control

After the traits have been recorded systematically, a total of 644 male birds were randomly selected for genotyping. These birds were 15 male parents and 629 male offspring. Of these 644 birds, 450 birds were genotyped with the 600 K Affymetrix^®^ Axiom^®^ HD genotyping array (Kranis et al., 2013), and the remaining 194 birds were genotyped with the Affy 55K array (Liu et al., 2019). The 600 K SNP chip contained 580,961 SNPs probes across 28 autosomes, two linkage groups (LGE64 and LGE22C19W28_E50C23), and two sex chromosomes. The 55 K SNP chip contained 52,184 SNP probes across 28 autosomes and a sex chromosome (chrZ). After converting the genome coordinates to a chicken reference genome (galGal5), 28 autosomes and a sex chromosome (chrZ) were extracted for further analysis. In the process of quality control of genotype, SNPs with minor allele frequency (MAF) of more than 0.5%, genotyping call rate of more than 97%, and Hardy–Weinberg equilibrium test *P*-value of more than 1 × 10^–6^ were retained. Finally, 547,020 and 51,984 SNPs were left for the 600 K and the 55 K chip data, respectively. In addition, a total of 23,213 SNPs was shared between the 600 K and the 55 K SNP chips.

Genotype imputation was performed with a two-step approach from 55 to 600 K and then imputed to WGS. Before the genotype imputation, pre-phasing was executed in Beagle 4.1 with default parameter (Browning and Browning, 2016). Firstly, using 450 birds with 600 K chip data as a reference panel, these 194 birds were imputed from 55 to 600 K chip data using Beagle 4.0 with pedigree and then merged with the 600 K chip data of these 194 birds and 450 birds using VCFtools. Secondly, all of the 644 birds with 600 K chip data were imputed to WGS data using a combined reference panel Beagle 4.1 with default parameter. The combined reference panels included 24 key individuals from the yellow-feather dwarf broiler population and 311 birds with WGS data from diverse chicken breeds. These 24 key individuals were selected by maximizing the expected genetic relationships (Ye et al., 2018). These 311 birds were downloaded from 10 BioProjects in ENA or NCBI. The combined reference panels contained 36,840,795 SNPs probes across 28 autosomes and a sex chromosome (chrZ). More detailed information could be found in our previous study (Ye et al., 2019).

After the genotype imputation was performed, the quality control of the imputed WGS data was conducted using PLINK v1.90b4.3 (Purcell et al., 2007) with the criteria of SNP call rate > 95%, individual call rate > 97%, MAF > 0.5%, and Hardy–Weinberg equilibrium *P*-value > 1.0e-6. In addition, individuals with existing Mendelian errors would be excluded. Finally, the remaining 626 individuals and 11,173,020 SNPs were used for further analysis.

### Genetic Parameter Estimations

The genomic heritability was calculated using the average information restricted maximum likelihood (AI-REML) method implemented in the software DMU v6.0 (Madsen et al., 2014). The statistical model was:

y=X⁢b+Z⁢g+e

where **y** is a vector of phenotypic values of all individuals, **b** is the vector of fixed effects including batch effect, and *g* was the vector of the animal additive genetic effect [$g∼N\,\left(0,\sigma_{g}^{2}\,G\right),\sigma_{g}^{2}$ is the additive genetic variance, where **G** is the marker-based genomic relationship matrix], **e** is the residual term [$e∼N\,\left(0,\sigma_{e}^{2}\,I\right)$, $\sigma_{e}^{2}$ is the residual variance and **I** was an identity matrix], and **X** and **Z** are incidence matrices relating the fixed effects and the additive genetic values to the phenotypic records; *G* is calculated using 600 K chip data as follows (VanRaden, 2008):

$$G=\frac{M\,M^{\text{T}}}{2\,\sum_{i=1}^{m}p_{i}\,\left(1-p_{i}\right)}$$

where *M* is a matrix of centered genotypes, *m* is the number of markers, and *p*_*i*_ is the minor allele frequency of SNP *i*.

### Genome-Wide Association Analyses Using Imputed WGS Data

Before GWAS was performed, the population structure of this chicken population was calculated by PLINK. Slight population stratification was found (Supplementary Figure S1), so we added top five principal components as covariates into the GWAS model to adjust the population structure. The univariate tests of association were performed using a mixed model approach implemented in the GEMMA v0.98.1 software (Zhou and Stephens, 2014). All sequence variants after quality control were tested for associations. The model was:

y=X⁢b+Z⁢g+S⁢a+e

where **y** is a vector of phenotypic values of all individuals, **X** and **Z** are incidence matrices relating the fixed effects and the additive genetic values to the phenotypic records, **b** is the vector of fixed effects including batch effect and top five principal components, **g** is a vector of the genomic breeding values of all individuals, **a** is the additive effect of the candidate variants to be tested for association, *S* is a vector of the variants’ genotype indicator variable coded as 0, 1, or 2, and **e** is the residual term, $e∼N\,\left(0,\sigma_{e}^{2}\,I\right)$. Genomic breeding values were assumed to be distributed as $g∼N\,\left(0,\sigma_{g}^{2}\,G\right)$, where *G* is the standardized relatedness matrix calculated by GEMMA using 600 K chip data. The Wald test was applied to test the alternative hypotheses of each SNP in the univariate models. The variance contribution to additive genomic variance by a SNP was calculated as follows:$H_{S}^{2}=2\,p\,\left(1-p\right)\,\beta^{2}$, where $H_{S}^{2}$ is the additive genomic variance explained by a SNP, *p* is the allele frequency, and β is the SNP effect as estimated from the GWAS results.

The Manhattan plot and quantile–quantile plot (QQ plot) were generated by the qqman package (Turner, 2018) in R. The threshold of genome-wide significant *P*-values was adjusted based on the effective number of independent tests for Bonferroni method. The imputed WGS data was pruned to 1,082,126 independent SNPs using PLINK with the command (–indep-pairwise 25 5 0.2) for estimating the effective number of independent tests. Finally, the effective number of independent tests were set to 200,629, estimated by sampleM (Gao et al., 2008). Therefore, the genome-wide suggestive and significant *P*-values were 4.98 × 10^–6^ (1.00/200, 629) and 2.49 × 10^–7^ (0.05/200, 629), respectively. For evaluating the extent of the false positive signals of the GWAS results, a genomic inflation factor (λ) was calculated as the median of the resulting chi-squared test statistics divided by the expected median of the chi-squared distribution with one degree of freedom (i.e., 0.454). Haploview 4.1 software (Barrett et al., 2005) was used to analyze the linkage disequilibrium around the significant SNPs. For identifying the independent signals precisely, the most significant SNPs were added as covariates into the univariate models in step-wise conditional analyses. In addition, the gene information file of chicken was downloaded from Ensembl gene build 94, and candidate genes were annotated using the software SnpEff version 4.3t (Cingolani et al., 2012).

### Transcriptomic Analysis Identifies Differentially Expressed Genes Associated With RFI

Raw reads of four samples (sample45561 and sample46307 with high RFI and sample45012 and sample46732 with low RFI) were downloaded from our previous study (Xu et al., 2016). The raw reads were processed to clean reads by filtering the low-quality reads and adaptor dimers. Clean reads were mapped to the chicken reference genome (galGal5) using HISAT2 v2.0.5 with the default parameter. Then, the alignments were assembled into full and partial transcripts using StringTie, and the transcripts for each sample were quantified using the GAL5. Finally, differential gene expression analysis was made with Ballgown in R environment (Pertea et al., 2016). In this study, differentially expressed transcripts or genes were identified based on an adjusted *P*-value less than 0.05 (false discovery rate of 5%) and the absolute value of log2-transformed of fold change more than or equal to 1. Function and pathway enrichment was performed with the R language package [clusterProfiler (Yu et al., 2012)]. Using the Benjamini–Hochberg method, the *P*-values of the KEGG pathway and the GO terms were adjusted for multiple testing (Benjamini and Hochberg, 1995). An adjusted *P*-value less than 0.05 was set as significant. In addition, the genome annotation information file was downloaded from Ensembl gene build 94.

### Validation of Candidate Genes Based on Differentially Expressed Genes

The identification of candidate genes of lead SNPs from GWAS results was performed basing on their corresponding genomic positions. The candidate gene regions were defined as extended 50-kb flanking regions both upstream and downstream of the lead SNP position. If there are no genes in the candidate gene regions, the nearest genes both upstream and downstream of the lead SNP position were selected as the candidate genes. To identify the causal genes or quantitative trait loci (QTLs), the overlap genes or regions between the candidate genes from sequence-based GWAS and DEGs were compared.

### Significant SNPs Compared With Reported QTLs

To compare the results from sequence-based GWAS with reported QTLs, significant and suggestively significant SNPs were selected to compare with the QTLs. These QTLs, all of which affect ADG, ADFI, and RFI, were selected from the Animal QTLdb (Hu et al., 2007), respectively. QTLs closest to the significant SNPs were extracted.

## Results

### Basic Descriptive Statistics of Analyzed Traits and Genetic Parameter Estimations

To achieve the genetic improvement of feed efficiency in broiler chickens, three traits were considered for analysis, including ADG, ADFI, and RFI. The basic descriptive statistics of these traits are shown in Table 1. The Shapiro–Wilk statistic of these traits was closed to 1 and the approximate *P*-values were less than 0.054, which indicated compliance with Gaussian distribution. The standard deviation (SD) of RFI, ADG, and ADFI were 8.83, 4.87, and 14.36, respectively. In addition, the coefficient of variation (CV) was 13.22% for ADFI and 16.82% for ADG. Therefore, these traits showed substantial phenotypic variation (Table 1). Using the AI-REML method, estimates of heritability, phenotypic correlation, and genetic correlation among ADG, ADFI, and RFI were calculated and shown in Table 2. The results show that the heritability estimates of ADG, ADFI, and RFI were 0.29 (0.004), 0.37 (0.005), and 0.38 (0.004), respectively. Genetic parameter analyses have shown that ADFI was both positively and highly interrelated with ADG and RFI. In addition, RFI is poorly correlated with ADG both in genetic relationship and in phenotypic correlation.

**TABLE 2:** Genetic parameters of the analyzed traits estimated by DMU with 600 K chip data in chicken.

Trait	ADG	ADFI	RFI
ADG	0.29 (0.004)	0.65	− 0.01
ADFI	0.68 (0.09)	0.37 (0.005)	0.63
RFI	0.17 (0.16)	0.75 (0.07)	0.38 (0.004)

*ADG, average daily gain; ADFI, average daily feed intake; RFI, residual feed intake. These diagonal values (mean ± SE), lower triangles values (mean ± SE), and upper triangles values are heritability estimates, genetic correlations, and phenotypic correlations for the analyzed traits, respectively.*

### Imputed Sequence-Based GWAS for ADG

**FIGURE 1:**
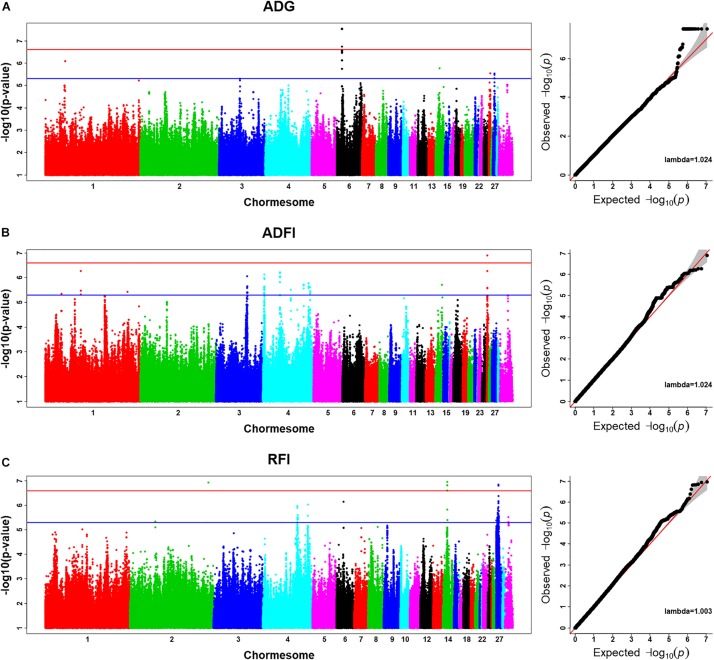
Manhattan plots and Q–Q plots of imputed sequence-based genome-wide association study (GWAS) for ADG **(A)**, ADFI **(B)**, and RFI **(C)**. The Manhattan plots indicate –log10(observed *P*-values) for markers (y-axis) against their corresponding position on each chromosome (x-axis), while the Q–Q plots show the expected –log10(*P*-values) vs. the observed –log10(*P*-values). The horizontal blue and red lines represent the genome-wide significant threshold (4.98 × 10^– 6^) and genome-wide suggestive significant threshold (2.49 × 10^– 7^), respectively. Lambda represents genomic inflation factor.

Using the univariate model, sequence-based GWAS was performed for ADG, and the Manhattan and QQ plots of GWAS results of ADG are shown in Figure 1A. Both 20 significant SNPs (Table 3) and 24 suggestive significant SNPs (Supplementary Table S1) associated with ADG were identified using the threshold of suggestive and significant *P*-values (4.98 × 10^–6^ and 2.49 × 10^–7^). These SNPs were located on these chromosomes (GGA1, GGA3, GGA6, GGA14, GGA25, and GGA27). All significant SNPs were in high linkage disequilibrium (Supplementary Figure S2) and located in a region that ranged from 535.4 to 538.9 kb on GGA6. After a stepwise conditional analysis, the *P*-value of significant or suggestive SNPs near the lead SNP would decrease below the suggestive threshold (Supplementary Figure S3). All significant SNPs were located in the intergenic region; the nearest gene was inositol polyphosphate multikinase (IPMK) (Table 3). Additionally, the genomic inflation factor of ADG was 1.024, which indicated that the results of the GWAS of ADG were acceptable.

**TABLE 3:** Information on significant SNPs associated with ADG, ADFI, and RFI.

Trait	SNPs	Chr.	Position	Allele	MAF	Bate (*SE*)	$H_{S}^{2}$	−log10(*P*-value)	Candidate or nearest genes	Annotation
ADG	6:5354190	6	5354190	T/G	0.008	−8.1 (1.44)	1.04	7.53	IPMK	Intergenic
ADG	rs731666382	6	5363742	T/C	0.008	−8.1 (1.44)	1.04	7.53	IPMK	Intergenic
ADG	rs731606971	6	5366469	G/C	0.008	−8.1 (1.44)	1.04	7.53	IPMK	Intergenic
ADG	rs733679573	6	5366491	T/C	0.008	−8.1 (1.44)	1.04	7.53	IPMK	Intergenic
ADG	rs315729276	6	5366673	G/T	0.008	−8.1 (1.44)	1.04	7.53	IPMK	Intergenic
ADG	rs732899645	6	5367289	T/G	0.008	−8.1 (1.44)	1.04	7.53	IPMK	Intergenic
ADG	6:5367311	6	5367311	T/G	0.008	−8.1 (1.44)	1.04	7.53	IPMK	Intergenic
ADG	rs315794025	6	5367445	G/T	0.008	−8.1 (1.44)	1.04	7.53	IPMK	Intergenic
ADG	rs739465741	6	5368822	G/C	0.008	−8.1 (1.44)	1.04	7.53	IPMK	Intergenic
ADG	rs731318403	6	5368918	A/T	0.008	−8.1 (1.44)	1.04	7.53	IPMK	Intergenic
ADG	6:5376152	6	5376152	T/A	0.008	−8.1 (1.44)	1.04	7.53	IPMK	Intergenic
ADG	6:5382202	6	5382202	T/C	0.008	−8.1 (1.44)	1.04	7.53	IPMK	Intergenic
ADG	rs731882620	6	5386041	G/T	0.009	−7.32 (1.39)	0.96	6.74	IPMK	Intergenic
ADG	6:5386085	6	5386085	A/G	0.008	−8.1 (1.44)	1.04	7.53	IPMK	Intergenic
ADG	6:5388772	6	5388772	C/G	0.008	−8.1 (1.44)	1.04	7.53	IPMK	Intergenic
ADG	rs736396454	6	5389064	A/G	0.008	−8.1 (1.44)	1.04	7.53	IPMK	Intergenic
ADG	6:5389116	6	5389116	A/G	0.008	−8.1 (1.44)	1.04	7.53	IPMK	Intergenic
ADG	6:5389139	6	5389139	G/A	0.008	−8.1 (1.44)	1.04	7.53	IPMK	Intergenic
ADG	rs732888314	6	5389223	C/T	0.008	−8.1 (1.44)	1.04	7.53	IPMK	Intergenic
ADG	rs741516185	6	5389362	G/A	0.008	−8.1 (1.44)	1.04	7.53	IPMK	Intergenic
ADFI	rs15997392	25	1147989	A/G	0.248	−5.42 (1.01)	10.96	6.9	COPA	Intron
**RFI**	rs313664593	2	1.39E + 08	A/G	0.014	−10.5 (1.96)	3.04	6.93	MTSS1	Intron
**RFI**	rs313288641	14	4767015	G/A	0.107	−4.41 (0.85)	3.72	6.61	FLCN COPS3	5 prime UTR Upstream
**RFI**	rs314690911	14	4779635	A/G	0.142	−4.04 (0.75)	3.98	6.96	COPS3 NT5M	Upstream Upstream
**RFI**	rs741733192	14	4782376	G/A	0.144	−3.98 (0.75)	3.91	6.82	COPS3 NT5M	Upstream Upstream
**RFI**	rs314351418	14	4782740	G/A	0.144	−3.98 (0.75)	3.91	6.82	COPS3 NT5M	Upstream Upstream
**RFI**	rs317155749	27	1212264	G/A	0.460	2.86 (0.54)	4.06	6.85	GTSF1	Intron
**RFI**	rs735238610	27	1220239	A/G	0.462	2.86 (0.54)	4.07	6.81	GTSF1	Intron

*ADG, average daily gain; ADFI, average daily feed intake; RFI, residual feed intake; $H_{S}^{2}$, the additive genomic variance explained by a SNP.*

### Imputed Sequence-Based GWAS for ADFI

Using the univariate model, sequence-based GWAS was performed for ADFI, and the Manhattan and QQ plots of the GWAS results of ADFI are shown in Figure 1B. Both one significant SNPs (Table 3) and 140 suggestive significant SNPs (Supplementary Table S1) associated with ADFI were identified using the threshold of suggestive and significant *P*-values (4.98 × 10^–6^ and 2.49 × 10^–7^). These SNPs were located on these chromosomes (GGA1, GGA3, GGA4, GGA14, and GGA25). The most significant SNPs were located at 1,147,989-bp position of GGA25. High linkage disequilibrium between SNPs of significant regions was found on GGA25 (Supplementary Figure S4). After a stepwise conditional analysis, the *P*-value of significant or suggestive SNPs near the lead SNP (rs15997392) would decrease below the suggestive threshold (Supplementary Figure S5). A total of seven genes were found in the region, which extended to 50-kb flanking regions both upstream and downstream of lead SNP (rs15997392) position (Supplementary Figure S5). The independent significant SNP (rs15997392) was an intron variant in coatomer protein complex subunit alpha (COPA) (Table 3). Additionally, the genomic inflation factor of ADFI was 1.024, which indicated that the results of the GWAS were acceptable.

### Imputed Sequence-Based GWAS for RFI

**FIGURE 2:**
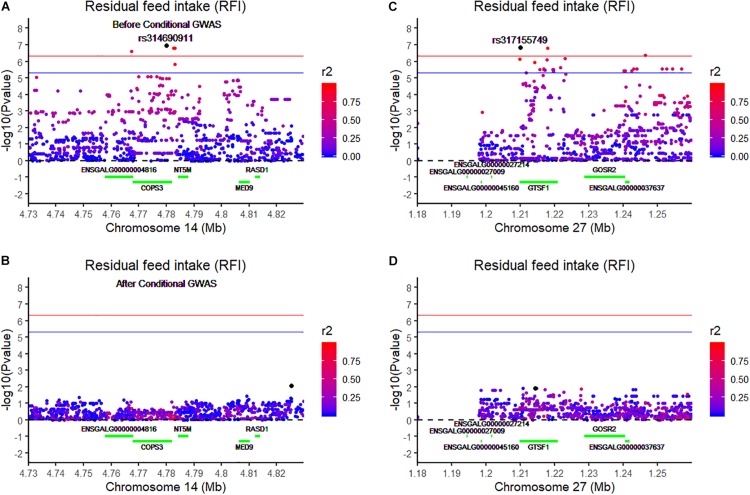
Regional association plot of the lead SNP associated with RFI at GGA14 and GGA27. The left panel of the figure shows the association results for RFI on chromosome 14 **(A)** before and **(B)** after conditional analysis on rs314690911. The right panel of the figure shows the association results for RFI on chromosome 27 **(C)** before and **(D)** after conditional analysis on rs317155749. The regional association plot indicates –log10(observed *P*-values) for markers (y-axis) against their corresponding position on each chromosome (x-axis). The horizontal blue and red lines represent the genome-wide significant threshold (4.98 × 10^– 6^) and genome-wide suggestive significant threshold (2.49 × 10^– 7^), respectively. The lead SNPs are denoted by a large black circle. The SNPs are represented by a colored circle according to the degree LD between the lead SNP. These green lines represent these genes located on this chromosome.

**FIGURE 3:**
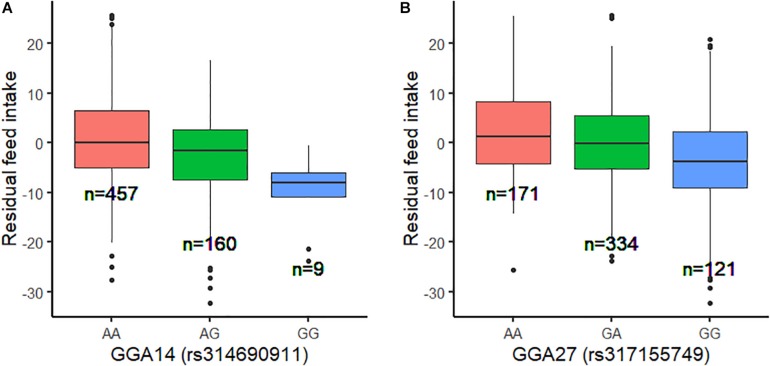
Genotype effect plot of lead SNP among three types at GGA14 and GGA27. **(A)** Genotype effect plot of lead SNP (rs314690911) among three types at GGA14. **(B)** Genotype effect plot of lead SNP (rs317155749) among three types at GGA27.

Using the univariate model, sequence-based GWAS was performed for RFI, and the Manhattan and QQ plots of the GWAS results of RFI are shown in Figure 1C. Both seven genome-wide significant SNPs (Table 3) and 100 suggestively significant SNPs (Supplementary Table S1) associated with RFI were identified using the threshold of suggestive and significant *P*-values (4.98 × 10^–6^ and 2.49 × 10^–7^). These significant SNPs were mainly located on these chromosomes (GGA2, GGA14, and GGA27). The most significant SNP was located at 4,779,635 bp on GGA14, which explained 5.46% of the phenotypic variance of RFI. Using SnpEff software, five candidate genes associated with RFI were identified and annotated (Table 3). These are MTSS I-BAR domain containing 1 (MTSS1), folliculin (FLCN), COP9 signalosome subunit 3 (COPS3), 5′,3′-nucleotidase, mitochondrial (NT5M), and gametocyte-specific factor 1 (GTSF1). After LD analysis, the high linkage disequilibrium between significant SNPs was found on GGA14 and GGA27 (Supplementary Figures S6, S7). To find the independent SNPs in a chromosome, a stepwise conditional analysis was performed by adding the lead SNP to the model as a fixed effect. The *P*-value of significant or suggestive SNPs near the lead SNP would decrease below the suggestive threshold (Figure 2A). Extended 50-kb flanking regions both upstream and downstream of the lead SNP position of the GWAS results in five genes [ENSGALG00000004816, COP93, NT5M, MED9 (mediator complex subunit 9), and ras-related dexamethasone-induced 1 (RASD1)] were indicated on GGA14 (Figure 2A) and six genes [ENSGALG00000027009, ENSGALG00000045610, ENSGALG00000027214, GTSF1, golgi SNAP receptor complex member 2 (GOSR2), and ENSGALG00000037637] on GGA27 (Figure 2B). In addition, the significant SNP of GGA2 was an isolated signal, which suggested that it may be a false positive significant site. Therefore, only two independent SNPs (rs314690911 and rs317155749) were suggested to have a significant association with RFI in this chicken population. Both substitution variants of rs314690911 (A to G) and rs317155749 (G to A) led to a significant decrease in the RFI phenotypic value (Figure 3). Additionally, the genomic inflation factor of RFI was 1.002, which is close to 1.00, reflecting that the results of GWAS were acceptable.

### Validation of Candidate Genes From GWAS Results Based on Differentially Expressed Genes

**FIGURE 4:**
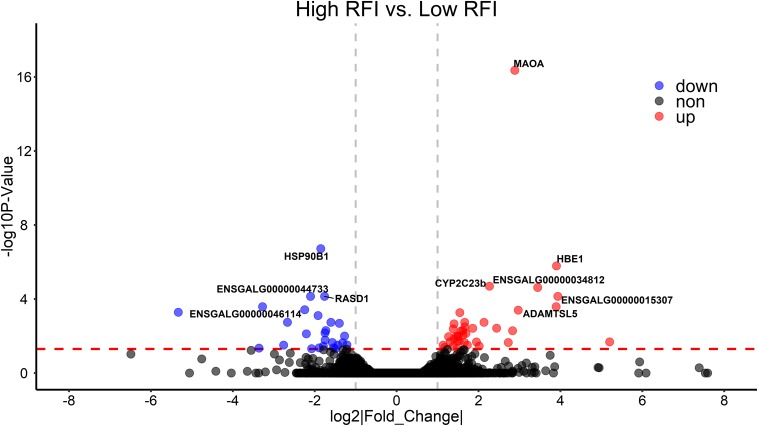
Differentially expressed genes (DEGs) between high- and low-RFI groups in chicken. The volcano plot indicates −log_10_ (observed *P*-values) for genes (y-axis) against their corresponding log2(|fold change|) of echo gene (x-axis). The horizontal red dotted line represents the significant threshold (0.05). The red, blue, and gray points represent up-regulated, down-regulated, and non-regulated genes in high-RFI groups, respectively.

To validate the list of candidate genes from the GWAS results, we performed transcriptomic analysis to identify DEGs between the high-RFI and low-RFI groups in chicken. There were 64 genes differentially expressed between the high-RFI and low-RFI groups with the gene expression fold change ranging from −5.33 to 5.20. Compared with the low-RFI group, both 38 up-regulated genes and 26 down-regulated genes were identified in the high-RFI group. All of the significant DEGs between the high-RFI and low-RFI groups are shown in Figure 4 and Supplementary Table S2, and the top 10 DEGs were monoamine oxidase A (MAOA), heat shock protein 90 beta family member 1 (HSP90B1), cytochrome P450 family 2 subfamily C polypeptide 23a (CYP2C23a), liver-enriched antimicrobial peptide 2 (LEAP2), RASD1, ABI family member 3 binding protein (ABI3BP), and ADAMTS like 5 (ADAMTSL5). After functional and pathway enrichment analysis, two molecular function (carbon–carbon lyase activity and unfolded protein binding) and one cellular component (endoplasmic reticulum lumen) categories were significantly enriched (Supplementary Table S3). Comparing the DEGs from the transcriptomic analysis (Figure 4) with these candidate genes that are located on the expanding 50-kb flanking regions both upstream and downstream of the independent SNPs rs314690911 and rs317155749 (Figure 2), RASD1 was the only one overlapped gene, which suggested a new QTL (GGA14:4767015–4882318) truly associated with RFI (Figure 2).

### Comparison Between Significant SNPs From GWAS and Reported QTLs

Single-nucleotide polymorphisms less than the threshold of the genome-wide significant *P*-values (4.98 × 10^–6^) were selected to compare with the reported QTLs. These QTLs were collected from the Animal QTL database based on their physical locations. For RFI, only two QTLs located on GGA4 and GGAZ were extracted, and the nearest distance of QTL between significant SNPs was 24,435,812 bp on GGAZ (Supplementary Table S4). For ADG, a total of four QTLs located on autosomes (GGA 1, 3, 6, and 27) were extracted, and the nearest distance of QTL between significant SNPs was 2,588,827 bp on GGA 27 (Supplementary Table S4). For ADFI, a total of six QTLs located on autosomes (GGA 1, 3, and 4) were extracted, and the nearest distance of QTL between significant SNPs was 1,504,353 bp on GGA 4 (Supplementary Table S4). No significant SNP located inside the reported QTL was found.

## Discussion

Feed efficiency plays an important role to improve profits and the environmental footprint in broiler production. The genetic dissection of RFI and its component traits would provide valuable insights for genetic improvement. In this study, a chicken population with imputed WGS data was used to perform GWAS to exploring the genetic mechanisms of feed efficiency. To the best of our knowledge, this is the first time that GWAS for RFI in chicken was performed using imputed sequence data, and transcriptomic analysis to identify DEGs between high-RFI and low-RFI groups in chicken was performed to validate these candidate genes.

### GWAS With Imputed Whole Genome Sequence Data

Recently, GWAS with imputed WGS data has been widely used in livestock species, especially in cattle (Daetwyler et al., 2014; Iso-Touru et al., 2016; Littlejohn et al., 2016). This is because genotype imputation would improve the power of GWAS (Marchini and Howie, 2010) and reduce the cost of genotyping. However, genotype imputation from SNP array to WGS data not only increased the marker density but also brought imputation error. Imputation errors will affect the probability of causal SNPs, which were determined by performing GWAS. Therefore, it is necessary to ensure high imputation accuracy before performing GWAS. To obtain higher imputation accuracy, genotype imputation with two-step approach was performed using a combined reference panel in this study. After ensuring quality control of the imputed WGS data, the average imputation accuracy (Beagle R2) of all SNPs was 0.871 ± 0.177 and ranged from 0.357 to 0.926 for different chromosomes (Supplementary Table S5). This high imputation accuracy was full enough to ensure the confidence of the GWAS results. This high imputation accuracy obtained may due to performing the imputation with a two-step approach (Kreiner-Moller et al., 2015) and using a combined reference panel (Ye et al., 2019). Compared with our previous study, which performed GWAS for RFI using 426 chickens with 600 K array data (Xu et al., 2016), new significant SNPs and genes were identified because more individuals (626 birds) and higher marker density (imputed WGS data) were utilized to perform GWAS. Although the power of GWAS was improved in this study, it is still difficult to pinpoint the causative variant because many significant SNPs existed with a high degree of linkage disequilibrium and had almost equally significantly associated variations, such as the significant SNPs of ADG on GGA6 (Table 3). Also, many markers’ effect were overlapped or overestimated due to LD (Table 3). Therefore, a stepwise conditional analysis was very necessary to find truly significant loci.

### Candidate Genes for Residual Feed Intake and Its Component Traits

After performing sequence-based GWAS, a lot of candidate genes were annotated as being associated with RFI and its component traits (Table 3 and Supplementary Table S1). For RFI, two independent significant SNPs (rs314690911 and SNP rs317155749) were identified via conditional GWAS. One was a variant upstream of COPS3, and the other is an intron variant of GTSF1. To our knowledge, the COPS3 gene was initially revealed to have a negative regulation on constitutive photomorphogenesis in *Arabidopsis thaliana*, which encodes the third subunit of the eight-subunit COP9 signalosome complex (Kwok et al., 1998). Also, COPS3 plays an important role in both tumorigenesis and progression. Previous research had shown that the amplification of COPS3 was strongly associated with a large tumor size (*P* = 0.0009) (Yan et al., 2007). The other is GTSF1, which came from the Uncharacterized Protein Family 0224 (UPF0224), could encode a 167-amino acid protein, and played an important role in liver cancer. Compared with those of the GTSF1-positive group, the sizes and the weights of the tumors of liver cancer in the GTSF1-negative group were decreased significantly (*P* < 0.05) (Gao et al., 2018). Moreover, RASD1 was the only one overlapped gene between the GWAS results and the DEGs between the high-RFI and the low-RFI groups. RASD1 is a highly conserved member of the Ras family of monomeric G proteins that was initially identified as a dexamethasone-inducible gene in AtT-20 mouse pituitary tumor cells (Kemppainen and Behrend, 1998). Vaidyanathan et al. (2004) indicate that RASD1 often promotes cell growth. Moreover, the current literature demonstrates that Rasd1 expression could be induced by a diversity of physiological stimuli and has many biological effects such as the regulation of circadian timekeeping, anxiety-related behavior, adipocyte differentiation, and hormone release (Bouchard-Cannon and Cheng, 2017). For ADG, all significant SNPs were located in the intergenic region, and IPMK was the nearest gene (Table 3). IPMK is a member of the IPK-superfamily of kinases, which plays an important role at the nexus of signaling, metabolic, and regulatory pathways (Kim et al., 2016). For example, IPMK is involved in the hypothalamic control of food intake via AMP-activated protein kinase signaling pathways (Lee et al., 2012). For ADFI, the most significant SNPs are located at 1147989-bp position of GGA25, which was an intron variant in COPA. COPA encodes the α-COP subunit of the coat protein I seven subunit complex that is involved with intracellular coated vesicle transport (Watkin et al., 2015). COPA mutations have recently been revealed to cause autoimmune interstitial lung, joint, and kidney disease (COPA syndrome). Moreover, the COP9 signalosome is a subunit of a highly conserved complex of COPS3. Therefore, we guess that the interaction of COPS3 and COP9 would impact on feed intake and further on RFI.

### Combined GWAS and Transcriptomic Analysis to Identify Candidate Genes

Nowadays, the combination of GWAS and transcriptomic analysis is an efficient method to identify the causal mutations of complex traits in livestock. In cattle, a previous study integrated RNA-Seq data and sequence-based GWAS data to explore the genetic mechanisms of mastitis resistance and milk production (Fang et al., 2017). In swine, using both the GWAS and the gene expression profile data, two genes (UBA domain containing 1 gene and Epsin 1 gene) were identified to be significantly associated with streptococcus Suis serotype 2 resistance (Ma et al., 2018). In chicken, integrating GAWS and transcriptome analysis, a new finding about the molecular mechanism underlying the formation of white/red earlobe color in chicken was revealed (Luo et al., 2018). In this study, combining imputed sequence-based GWAS and transcriptome analysis between the high-RFI and low-RFI groups, we also found an overlapped gene (RASD1) significantly associated with RFI (Figures 2, 4). This finding also suggested that imputed sequence-based GWAS was an efficient method to identify significant SNPs.

### Comparison Between GWAS Results and Reported QTLs

Comparing the GWAS results with the reported QTLs, we found no significant SNP located inside the reported QTL in this study (Supplementary Table S4). This is mainly due to the number of reported QTLs, associated with RFI, ADFI, and ADG, which are still very limited in QTLdb, especially for RFI. In QTLdb, only 40 QTLs were reported to be associated with RFI and 17 QTLs located in the range from 16433894 to 17287377 bp on GGA12. Moreover, the different genetic backgrounds also would result in different QTL regions.

## Conclusion

Using imputed sequence-based GWAS is an efficient method to identify significant SNPs and candidate genes in chicken. In this study, using imputed sequence-based GWAS, we identified seven significant SNPs associated with RFI, 20 significant SNPs associated with ADG, and one significant SNP associated with ADFI. Furthermore, by combining regional conditional GWAS and transcriptome analysis between the high-RFI and the low-RFI groups, an overlapped gene (RASD1) was identified to be associated with RFI, which also suggested that a new QTL (GGA14: 4767015–4882318) was truly associated with RFI. Our results provide valuable insights into the genetic mechanisms of RFI and component traits in chickens.

## Data Availability Statement

Raw reads of four samples (sample45561 and sample46307 with high RFI and sample45012 and sample46732 with low RFI) were downloaded from here: http://www.animalgenome.org/repository/pub/SCAU2016.0217/. The imputed WGS data of these 626 birds have been uploaded to the figshare repository (https://doi.org/10.6084/m9.figshare.10264913.v1).

## Ethics Statement

Animal care and experiments were conducted according to the Regulations for the Administration of Affairs Concerning Experimental Animals (Ministry of Science and Technology, China) and approved by the Animal Care and Use Committee of the South China Agricultural University, Guangzhou, China (approval number: SCAU#2014-10).

## Author Contributions

SY, ZZ, and JL conceived the study, designed the project, and helped in preparing the draft. XZ provided the chickens’ dataset. SY and RZ finished the genotype imputation and performed GWAS. SY and Z-TC performed the transcriptomic analysis. Z-TC, RZ, SD, JT, XY, HZ, and ZC participated in the design and contributed to the manuscript. All the authors read and approved the manuscript.

## Conflict of Interest

The authors declare that the research was conducted in the absence of any commercial or financial relationships that could be construed as a potential conflict of interest.
