# Supplementary material for: New Insights From Imputed Whole-Genome Sequence-Based Genome-Wide Association Analysis and Transcriptome Analysis: The Genetic Mechanisms Underlying Residual Feed Intake in Chickens
Source: Front Genet. 2020 Apr 3;11:243. doi: 10.3389/fgene.2020.00243 (PMC7147382; doi:10.3389/fgene.2020.00243)

**Figure S1. Principal component analysis of chicken population**


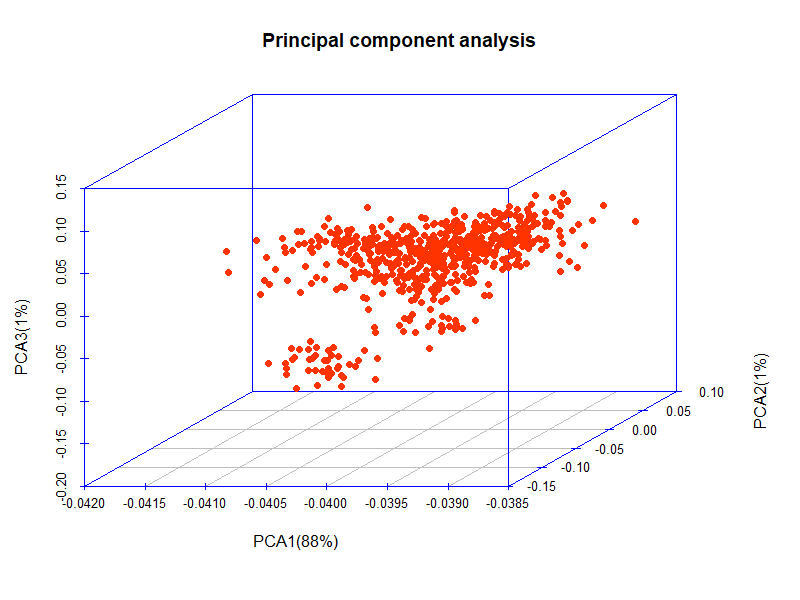


**Figure S2.** **LD analysis of** **the significant region on chromosome 6 of ADG.** The strong LD block is defined as D’ ≥ 0.8.


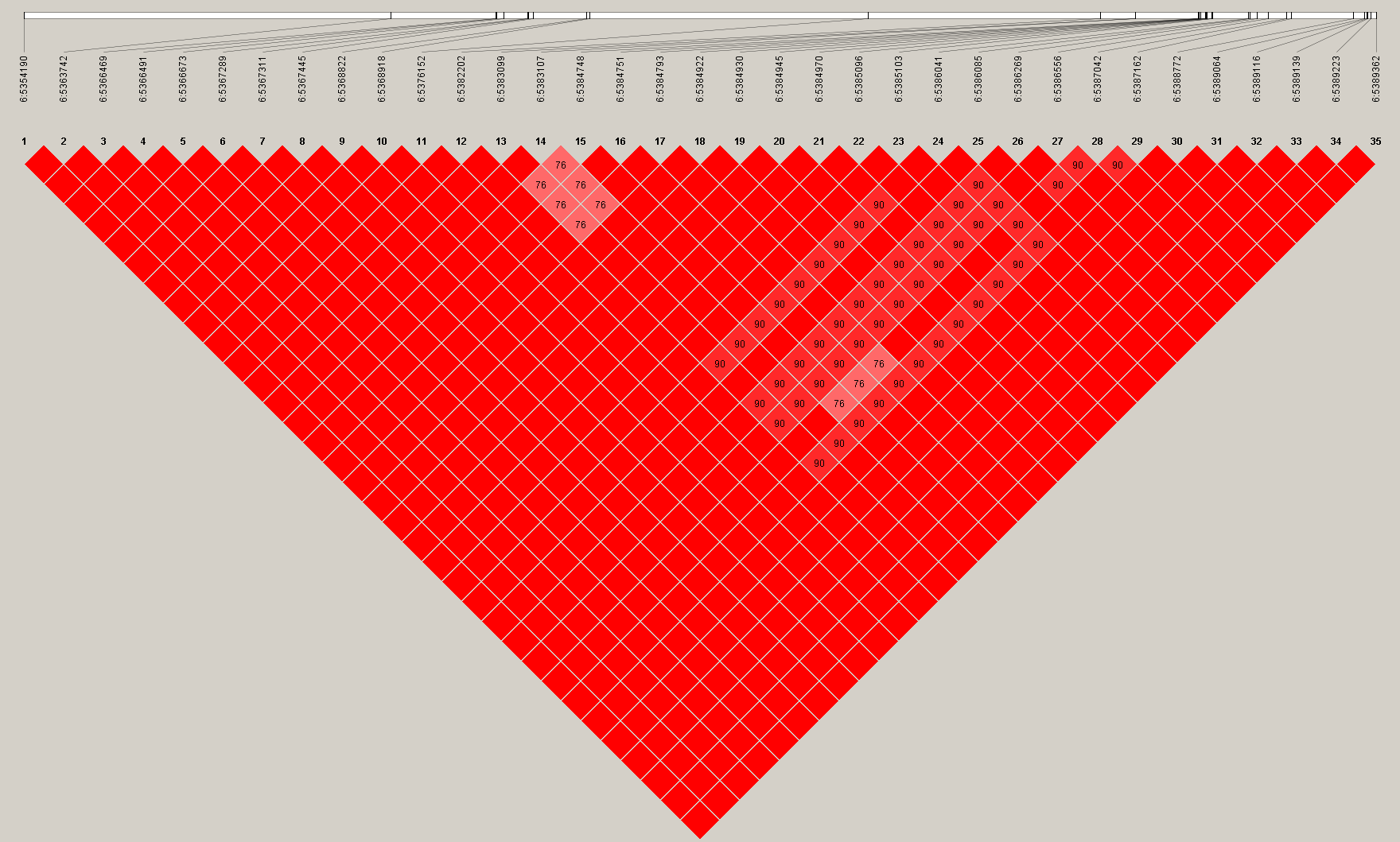


**Figure S3. Regional association plot of the lead SNP associated with ADG on GGA6.** The regional association plot indicates −log10 (observed P-values) for markers (y-axis) against their corresponding position on each chromosome (x-axis). The horizontal blue and red lines represent the genome-wide significant threshold (4.98 × 10−6) and genome-wide suggestive significant threshold (2.49 × 10−7), respectively. The lead SNPs are denoted by large black circle. SNPs are represented by colored circle according to the degree LD between the lead SNP.


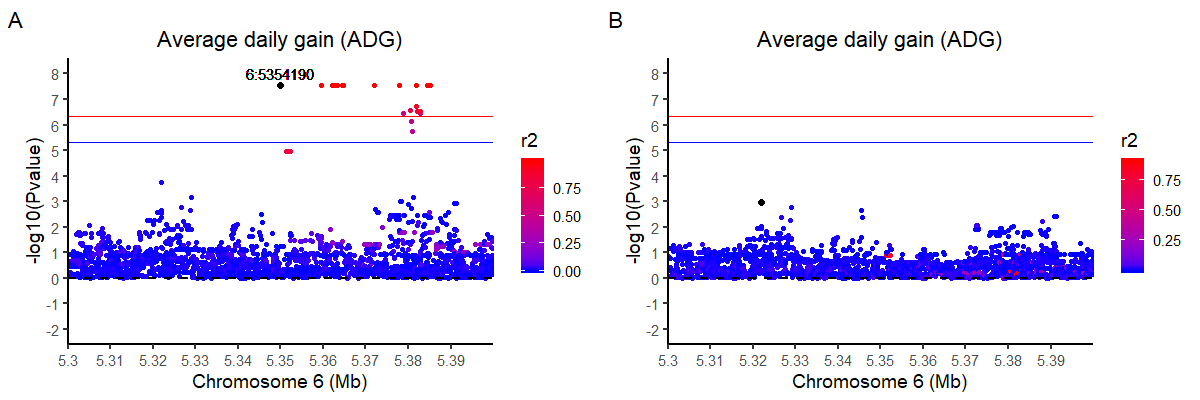


**Figure S4. LD analysis of the significant region on chromosome 25 of ADFI.** The strong LD block is defined as D’ ≥ 0.8.


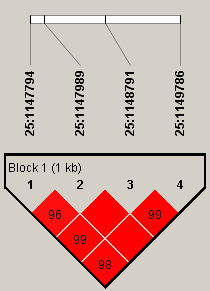


**Figure S5. Regional association plot of the lead SNP associated with ADFI on GGA25.** The regional association plot indicates −log10 (observed P-values) for markers (y-axis) against their corresponding position on each chromosome (x-axis). The horizontal blue and red lines represent the genome-wide significant threshold (4.98 × 10−6) and genome-wide suggestive significant threshold (2.49 × 10−7), respectively. The lead SNPs are denoted by large black circle. SNPs are represented by colored circle according to the degree LD between the lead SNP. These green lines represent these genes located on this chromosome.


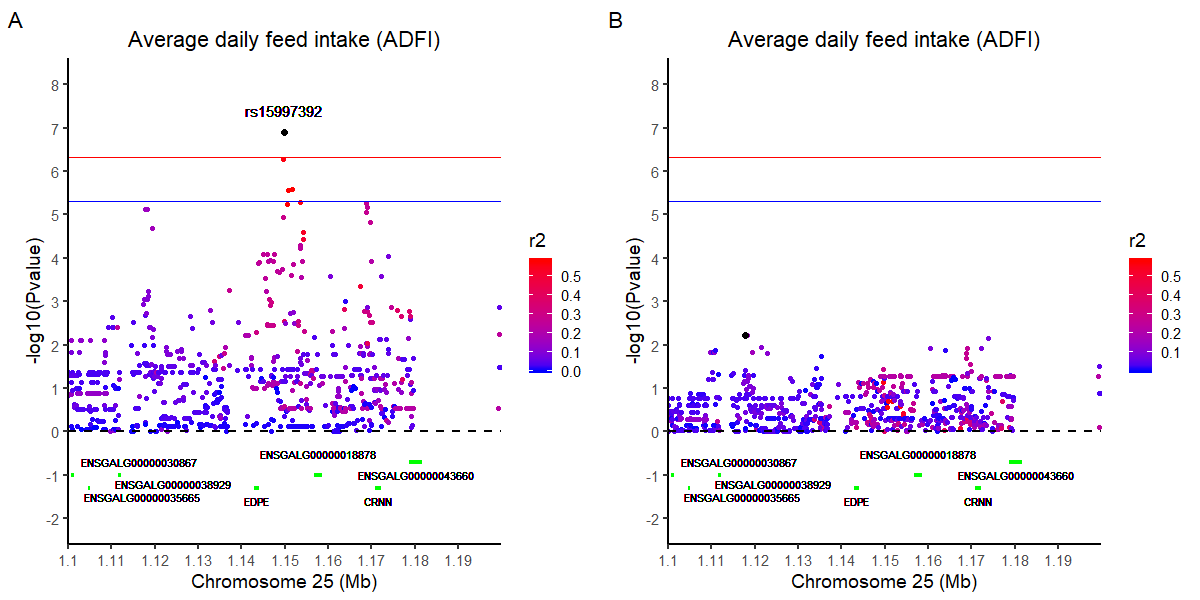


**Figure S6. LD analysis of the significant region of RFI on chromosome 14.** The strong LD block is defined as D’ ≥ 0.8.


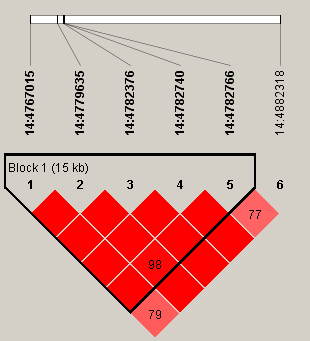


**Figure S7. LD analysis of the significant region of RFI on chromosome 27.** The strong LD block is defined as D’ ≥ 0.8.


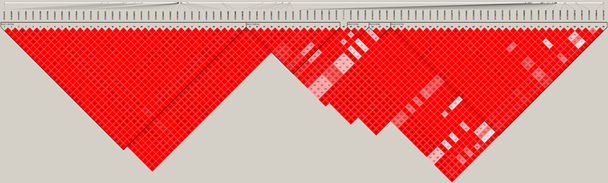

Supplement: Supplementary file 1 [file Data_Sheet_1.docx]
